# The Difference and Significance of Parietal Pleura Invasion and Rib Invasion in Pathological T Classification With Non-Small Cell Lung Cancer

**DOI:** 10.3389/fonc.2022.878482

**Published:** 2022-04-28

**Authors:** Lei-Lei Wu, Chong-Wu Li, Kun Li, Li-Hong Qiu, Shu-Quan Xu, Wei-Kang Lin, Guo-Wei Ma, Zhi-Xin Li, Dong Xie

**Affiliations:** ^1^ Department of Thoracic Surgery, Shanghai Pulmonary Hospital, School of Medicine, Tongji University, Shanghai, China; ^2^ Department of Thoracic Surgery, Sun Yat-sen University Cancer Center, State Key Laboratory of Oncology in South China, Collaborative Innovation Center for Cancer Medicine, Guangzhou, China; ^3^ School of Medicine, Tongji University, Shanghai, China

**Keywords:** parietal pleura invasion, rib invasion, T classification, survival, upstage

## Abstract

**Objective:**

This study was to explore the difference and significance of parietal pleura invasion and rib invasion in pathological T classification with non-small cell lung cancer.

**Methods:**

A total of 8681 patients after lung resection were selected to perform analyses. Multivariable Cox analysis was used to identify the mortality differences in patients between parietal pleura invasion and rib invasion. Eligible patients with chest wall invasion were re-categorized according to the prognosis. Cancer-specific survival curves for different pathological T (pT) classifications were presented.

**Results:**

There were 466 patients considered parietal pleura invasion, and 237 patients served as rib invasion. Cases with rib invasion had poorer survival than those with the invasion of parietal pleura (adjusted hazard ratio [HR]= 1.627, *P* =0.004). In the cohort for parietal pleura invasion, patients with tumor size ≤5cm reached more satisfactory survival outcomes than patients with tumor size >5cm (unadjusted HR =1.598, *P* =0.006). However, there was no predictive difference in the cohort of rib invasion. The results of the multivariable analysis revealed that the mortality with parietal pleura invasion plus tumor size ≤5cm were similar to patients with classification pT3 (*P* =0.761), and patients for parietal pleura invasion plus tumor size >5cm and pT4 had no stratified survival outcome (P =0.809). Patients identified as rib invasion had a poorer prognosis than patients for pT4 (*P* =0.037).

**Conclusions:**

Rib invasion has a poorer prognosis than pT4. Patients with parietal pleura invasion and tumor size with 5.1-7.0cm could be appropriately up-classified from pT3 to pT4.

## Introduction

Lung cancer is the second most common malignancy in the global cancer spectrum of morbidity and is still the leading cause of cancer mortality (1). The prognosis of lung cancer is poor, and the 2-year overall survival rate of patients with non-small cell lung cancer (NSCLC) and small cell lung cancer is approximately 42% and 15%, respectively (2). The eighth edition of the tumor-lymph node-metastasis (TNM) classification system from the American Joint Committee on Cancer was launched in January 2017, the most accurate and newest classification system (3, 4). However, the role played by some uncommon factors that may be related to T classification in prognosis and the issue of ascending T classification are still not annotated in enough detail in National Comprehensive Cancer Network guidelines and clinical practice (4–6). Precise evaluation of T descriptor plays a crucial role in estimating prognosis and deciding on the most appropriate treatment. Nevertheless, the prognostic difference among parietal pleura invasion, rib invasion, and other established T classifications is still unclear (6).

Previous studies suggested that patients with parietal pleura invasion with tumor size ≤7cm had a close survival rate to patients with T3 descriptor (7, 8). Nevertheless, the results from other studies revealed that the patients with parietal pleura invasion or other chest wall invasion reached worse long-term survival outcomes than other patients in the group for classification T3 or T4 (9, 10). Thus, the prognosis of parietal pleura invasion and rib invasion remains unclear because of the unclear T classification. Besides, it is still inconsistent in view on whether there is a prognostic difference between patients with rib invasion and parietal pleural invasion (8, 11). Therefore, it is important to investigate the prognostic difference and significance of parietal pleura invasion and rib invasion in pathological T (pT) classification with NSCLC. This study aimed to elucidate the significance of the above two factors for survival and their risk grade in pT-classification when combined with different-category tumor sizes.

## Methods

### Patients

**Figure 1:**
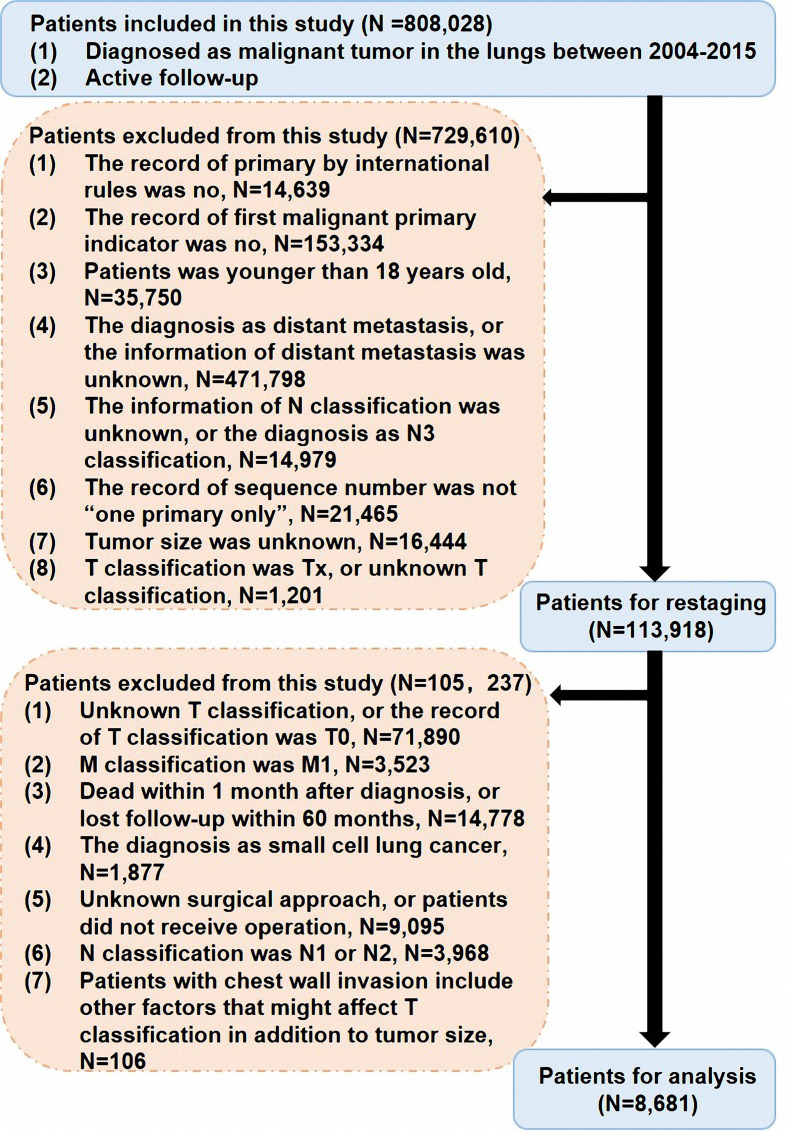
The flow chart of this study.

Cases were diagnosed as NSCLC in the Surveillance, Epidemiology, and End Results (SEER) database, which provides data that does not identify patients, healthcare providers, or hospitals. This database contains clinicopathological and survival data of cancer patients from 18 registries. All patient records were anonymized before analysis. Institutional review board approval was waived by the Ethics Committee of Shanghai Pulmonary Hospital. Patients who were histologically diagnosed with NSCLC in the lungs as their first primary malignancy from 2004 to 2015 were recruited. The selection criteria of patients are presented in **Figure 1**. The data for a total of 8681 patients were used to perform the main analysis. Information collected from the SEER database included race/ethnicity, sex, age at diagnosis, the approach of treatment (including surgical treatment, radiotherapy, and chemotherapy), tumor size, tumor differentiation, histological subtype, pathological TNM stage, marital status, tumor location, survival time, and cause on disease.

### Follow-up

Follow-up duration ranged from 1.0 to 155.0 months, with a median of 44.0 months. Those patients had definitive survival status, death or alive. Cancer-specific survival (CSS), which was the duration from the date of diagnosis to death caused by lung cancer, was regarded as our observational endpoint.

### Statistical Analysis

Pearson’s χ ^2^ (2) statistic method and Fisher exact test were used to estimate the differences in the distribution of each categorical variable between groups. The continuous numerical variable was tested by the Mann-Whitney U test if it did not conform to a normal distribution. Hazard ratios (HRs) and 95% confidence intervals (CIs) were calculated using univariable and multivariable Cox regression analyses, respectively (method was Enter selection). The average value of each covariate was calculated by the multivariable Cox regression model and estimated the adjusted survival curves of different categorical variables. Kaplan-Meier survival analysis was used to draw the survival curves. Statistical tests were considered statistically significant with a two-sided *P* value <0.05. All statistical analyses were performed using SPSS statistics 25.0 software (IBM SPSS, Inc., Chicago, IL, USA).

## Results

### Patient Characteristics

In the cohort, ages ranged from 18 years to 96 years old (median was 68 years old). The majority of the patients were Caucasian (N =7296, 84%), and 4517 (52%) were male patients. Four hundred and sixty-six patients were considered as parietal pleura invasion, and 237 patients were considered as rib invasion. Two hundred thirty-seven patients had rib invasion, including 25 patients (10.5%) with tumor size ≤3cm, 73 patients (30.8%) with tumor size 3-5cm, 76 patients (32.1%) with tumor size 5-7cm, and the remaining patients with tumor size >7cm. Among cases with only parietal pleural invasion and no rib invasion, there were 60 patients with tumor size of ≤3cm, 82 patients with tumor size of 3-5cm, 53 patients with tumor size 5-7cm, and 34 patients with tumor size of >7cm. The other detailed information on patient characteristics is shown in **Table 1**.

**Table 1:** The clinicopathological characteristics for patients with different status of parietal pleura invasion and rib invasion.

	All	Parietal pleura invasion			Rib invasion		
	(N = 8,681)	No (N = 8,215)	Yes (N =466)		No (N = 8,444)	Yes (N = 237)	
Variables	No. of patients (%)/Mean ± SD			*P* value	No. of patients (%)/Mean ± SD		*P* value
**Sex**				<0.001^*^			<0.001^*^
Male	4,517 (52.0)	4,234 (51.5)	283 (60.7)		4,367 (51.7)	150 (63.3)	
Female	4,164 (48.0)	3,981 (48.5)	183 (39.3)		4,077 (48.3)	87 (36.7)	
**Race/ethnicity**				0.640^**^			0.539^**^
Caucasians	7,296 (84.0)	6,903 (84.0)	393 (84.3)		7,095 (84.0)	201 (84.8)	
Black	792 (9.2)	745 (9.1)	47 (10.1)		767 (9.1)	25 (10.5)	
Other	575 (6.6)	549 (6.7)	26 (5.6)		564 (6.7)	11 (4.7)	
Unknown	18 (0.2)	18 (0.2)	0 (0.0)		18 (0.2)	0 (0.0)	
**Age (year)**	67.8 ± 10.1	67.8 ± 10.6	65.7 ± 9.9	<0.001^***^	67.9 ± 10.1	64.7 ± 10.1	<0.001^***^
**Tumor size (cm)**				<0.001^*^			<0.001^*^
≤3	4,965 (57.2)	4,880 (59.4)	85 (18.2)		4,940 (58.5)	25 (10.5)	
3-5	1,671 (19.2)	1,516 (18.5)	155 (33.3)		1,598 (18.9)	73 (30.8)	
5-7	666 (7.7)	537 (6.5)	129 (27.7)		590 (7.0)	76 (32.1)	
>7	1,379 (15.9)	1,282 (15.6)	97 (20.8)		1,316 (15.6)	63 (26.6)	
**Differentiation grade**				<0.001^*^			<0.001^**^
Grade I	1,374 (15.8)	1,363 (16.6)	11 (2.4)		1,370 (16.2)	4 (1.7)	
Grade II	3,579 (41.2)	3,424 (41.7)	155 (33.3)		3,518 (41.7)	61 (25.7)	
Grade III	3,077 (35.5)	2,833 (34.5)	244 (52.3)		2,941 (34.8)	136 (57.4)	
Grade IV	194 (2.2)	164 (2.0)	30 (6.4)		174 (2.1)	20 (8.4)	
Unknown	457 (5.3)	431 (5.2)	26 (5.6)		441 (5.2)	16 (6.8)	
**Histological type**				<0.001^*^			<0.001^*^
Adenocarcinoma	4,633 (53.4)	4,486 (54.6)	147 (31.5)		4,571 (54.1)	62 (26.2)	
Squamous cell	2,668 (30.7)	2,440 (29.7)	228 (48.9)		2,548 (30.2)	120 (50.5)	
Other	1,112 (12.8)	1,042 (12.7)	70 (15.1)		1,073 (12.7)	39 (16.5)	
Unknown NSCLC	268 (3.1)	247 (3.0)	21 (4.5)		252 (3.0)	16 (6.8)	
**Surgical type**				0.001^*^			<0.001^*^
Limited resection	1,561 (18.0)	1,508 (18.4)	53 (11.4)		1,544 (18.3)	17 (7.2)	
Lobectomy	6,724 (77.5)	6,335 (77.1)	389 (83.5)		6,513 (77.1)	211 (89.0)	
Pneumonectomy	396 (4.5)	372 (4.5)	24 (5.1)		387 (4.6)	9 (3.8)	
**Chemotherapy**				<0.001^*^			<0.001^*^
No	7,159 (82.5)	6,930 (84.4)	229 (49.1)		7,057 (83.6)	102 (43.0)	
Yes	1,522 (17.5)	1,285 (15.6)	237 (50.9)		1,387 (16.4)	135 (57.0)	
**Radiotherapy**				<0.001^*^			<0.001^*^
No	7,931 (91.4)	7,672 (93.4)	259 (55.6)		7,815 (92.5)	116 (48.9)	
Before surgery	162 (1.9)	110 (1.3)	52 (11.2)		119 (1.4)	43 (18.1)	
After surgery	550 (6.3)	407 (5.0)	143 (30.7)		479 (5.7)	71 (30.0)	
Other	38 (0.4)	26 (0.3)	12 (2.5)		31 (0.4)	7 (3.0)	
**Tumor Location**				<0.001^**^			<0.001^**^
Upper	5,145 (59.3)	4,775 (58.1)	370 (79.4)		4,948 (58.6)	197 (83.1)	
Middle	431 (5.0)	427 (5.2)	4 (0.9)		428 (5.1)	3 (1.3)	
Lower	2,805 (32.3)	2,735 (33.3)	70 (15.0)		2,780 (32.9)	25 (10.5)	
Other	203 (2.3)	187 (2.3)	16 (3.4)		195 (2.3)	8 (3.4)	
Unknown	97 (1.1)	91 (1.1)	6 (1.3)		93 (1.1)	4 (1.7)	
**Marital status**				0.02^*^			0.146^**^
Unmarried	3,483 (40.1)	3,300 (40.2)	183 (39.3)		3,384 (40.1)	99 (41.8)	
Married	4,893 (56.4)	4,616 (56.2)	277 (59.4)		4,758 (56.3)	135 (57.0)	
Unknown	305 (3.5)	299 (3.6)	6 (1.3)		302 (3.6)	3 (1.3)	
**Year of diagnosis**				<0.001^*^			<0.001^*^
2004-2009	968 (11.2)	826 (10.1)	142 (30.5)		826 (9.8)	142 (59.9)	
2010-2015	7,713 (88.8)	7,389 (89.9)	324 (69.5)		7,618 (90.2)	95 (40.1)	

SD, standard deviation; NSCLC, non-small-cell lung cancer.

^*^Pearson’s χ^2^ (2) statistic method was performed in those variables.

^**^These variables were calculated by Fisher exact test.

^***^Age as a continuous numerical variable was used Mann-Whitney U test, because it did not conform to a normal distribution.

### Prognostic Significance in Chest-Wall Invasion

**Figure 2:**
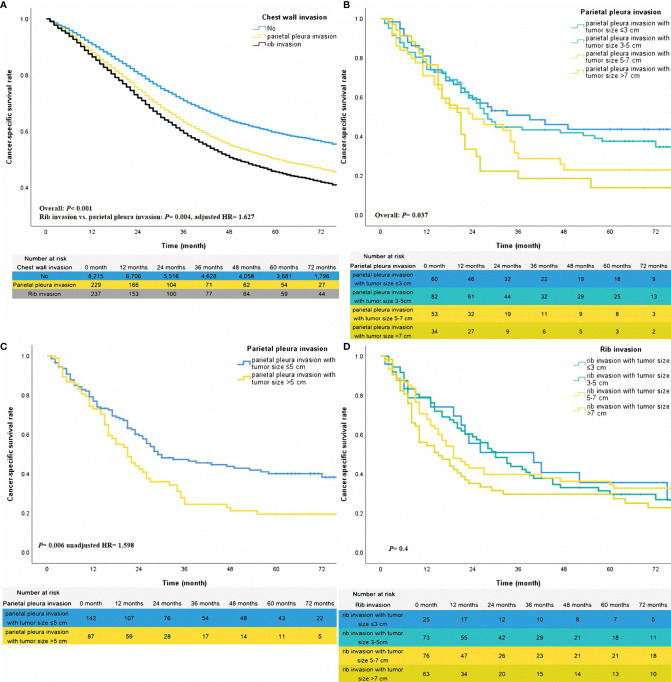
The adjusted survival curves for different statuses of chest wall invasion **(A)**. The unadjusted survival curves for different classifications of parietal pleura invasion **(B, C)** and rib invasion **(D)**.

In this cohort, 5250 death events occurred in the 8681 NSCLCs. For the patients with rib invasion, 200 deaths occurred out of 237 patients. Among 229 cases of only parietal pleural invasion without rib invasion, there were 177 death events. The median survival time for patients without chest wall invasion, or with only parietal pleural invasion, or with rib invasion was 46 months, 21 months, and 19 months, respectively. Patients without chest wall invasion showed better survival than other patients (**Table 2**, all *P <*0.05). Besides, the prognosis in patients with rib invasion was poorer than in patients with only parietal pleural invasion and no rib invasion (**Figure 2A**, adjusted HR=1.627, *P* =0.004). The 3-year and 5-year CSS rates for patients with different levels of chest wall invasion were 64% vs. 56% (without chest wall invasion), 37% vs. 32% (only parietal pleural invasion and no rib invasion), and 34% vs. 22% (rib invasion), respectively. In addition, sex, tumor differentiation, tumor location for lower lobe, age, other histological subtypes of NSCLCs, other races, lobectomy tumor size, year of diagnosis, radiotherapy, and chest wall invasion (parietal pleura invasion vs. no, adjusted HR= 1.400, 95% CI 1.167-1.678, rib invasion vs. no, adjusted HR =1.620, 95% CI 1.351-1.944) were identified as independent prognostic factors (**Table 2**).

**Table 2:** Univariable and multivariable Cox regression analysis for cancer-specific mortality in patients with different statuses of parietal pleura invasion and rib invasion.

	Univariable analysis			Multivariable analysis		
Variables	HR	95% CI	P-Value	HR	95% CI	P-Value
**Sex**						
Male	1	reference		1	reference	
Female	0.682	0.637-0.731	<0.001	0.786	0.732-0.844	<0.001
**Tumor differentiation**						
Grade I	1	reference		1	reference	
Grade II	1.827	1.611-2.071	<0.001	1.749	1.538-1.988	<0.001
Grade III	2.828	2.498-3.200	<0.001	2.336	2.051-2.660	<0.001
Grade IV	2.991	2.373-3.770	<0.001	1.855	1.459-2.360	<0.001
Unknown	2.313	1.931-2.770	<0.001	1.888	1.570-2.269	<0.001
**Tumor location**						
Upper lobe	1	reference		1	reference	
Middle lobe	0.888	0.751-1.050	0.164	1.087	0.918-1.286	0.335
Lower lobe	1.108	1.029-1.193	0.007	1.158	1.073-1.250	<0.001
Other location	1.810	1.495-2.193	<0.001	1.308	1.071-1.598	0.009
Unknown	1.413	1.041-1.918	0.027	1.381	1.015-1.880	0.040
**Age (median, year)**						
≤68	1	reference		1	reference	
>68	1.310	1.223-1.403	<0.001	1.451	1.349-1.560	<0.001
**Histological subtypes**						
Adenocarcinoma	1	reference		1	reference	
Squamous cell carcinoma	1.498	1.386-1.619	<0.001	0.997	0.899-1.061	0.574
Other NSCLCs	1.653	1.496-1.827	<0.001	1.311	1.183-1.453	<0.001
Unknown NSCLC	1.702	1.425-2.032	<0.001	0.983	0.817-1.181	0.851
**Chemotherapy**						
No	1	reference		1	reference	
Yes	1.646	1.521-1.781	<0.001	0.920	0.835-1.013	0.091
**Radiotherapy**						
No	1	reference		1	reference	
Before surgery	2.136	1.754-2.600	<0.001	1.489	1.198-1.852	<0.001
After surgery	2.369	2.123-2.643	<0.001	1.660	1.470-1.875	<0.001
Unknown	1.917	1.260-2.916	0.002	1.199	0.783-1.838	0.404
**Marital status**						
Non-married	1	reference				
Married	0.933	0.869-1.001	0.052			
Unknown	0.838	0.685-1.025	0.085			
**Race/ethnicity**						
Caucasians	1	reference		1	reference	
Black	1.047	0.933-1.176	0.434	1.057	0.941-1.188	0.352
Other	0.811	0.701-0.938	0.005	0.870	0.752-1.007	0.062
Unknown	0.490	0.184-1.306	0.154	0.677	0.254-1.806	0.436
**Surgical treatment**						
Limited resection	1	reference		1	reference	
Lobectomy	0.864	0.791-0.943	0.001	0.708	0.645-0.777	<0.001
Pneumonectomy	1.547	1.321-1.811	<0.001	0.752	0.631-0.896	0.001
**Tumor size**						
≤3cm	1	reference		1	reference	
3-5cm	1.763	1.611-1.930	<0.001	1.594	1.449-1.753	<0.001
5-7cm	2.441	2.168-2.748	<0.001	2.091	1.836-2.381	<0.001
>7cm	2.894	2.655-3.155	<0.001	3.000	2.650-3.397	<0.001
**Chest wall invasion**						
No	1	reference		1	reference	
Parietal pleura invasion	2.144	1.805-2.546	<0.001	1.400	1.167-1.678	<0.001
Rib invasion	2.432	2.070-2.856	<0.001	1.620	1.351-1.944	<0.001
**Year of diagnosis**						
2004-2009	1	reference		1	reference	
2010-2015	0.529	0.483-0.580	<0.001	1.310	1.155-1.487	<0.001

HR, hazard ratio; CI, confidence interval; NSCLC, non-small cell lung carcinoma.

Cox regression’s method was Enter selection.

### Stratified Effect for Tumor Size

To further investigate the stratified effect of tumor size on patients with different statuses of chest wall invasion, the sub-group analysis of patients with rib invasion or only parietal pleura invasion was performed. The method of sub-group analysis was Kaplan-Meier. In the patients with only parietal pleura invasion, cases with tumor size ≤5cm might reach a better survival trend over those with tumor size >5cm (**Figure 2B**, overall *P* =0.037). Thus, we re-categorized groups with tumor sizes ≤5cm and >5cm into one group. According to this classification, sub-groups for tumor size ≤5cm and tumor size >5cm got a meaningful stratification effect (**Figure 2C**, unadjusted HR =1.598, *P* =0.006). Given different levels of tumor size did not have a prognostic impact on patients of rib invasion (**Figure 2D**, overall P =0.4). Therefore, those patients were grouped together.

### Survival Analysis of T Classification

**Figure 3:**
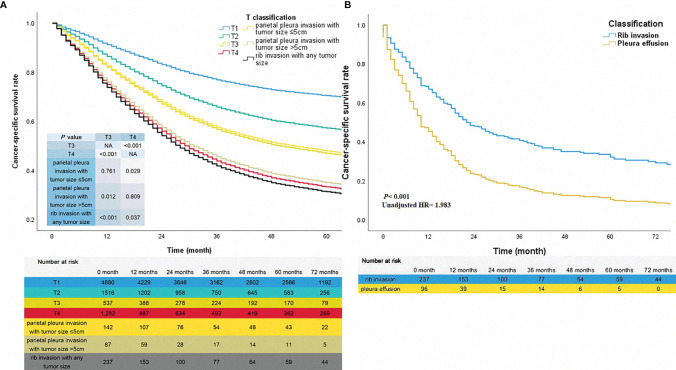
The adjusted survival curves of different pathological T classifications and chest wall invasion **(A)**. The unadjusted survival curves for pleura effusion (TxN0M1a) and rib invasion **(B)**.

The three classifications mentioned above, plus the classifications pT1, pT2, pT3, and pT4 that originally existed were conducted for survival analysis together to determine the prognostic outcomes of chest wall invasion with different tumor sizes in the pT classification. The results of adjusted survival curves confirmed that patients with rib invasion had the worst survival in that cohort (**Table 3** and **Figure 3A**, rib invasion vs. T4, P =0.037). Parietal pleura invasion with tumor size ≤5cm, parietal pleura invasion with tumor size >5cm, and rib invasion with any tumor size were confirmed as independent prognostic risk indictors (**Table 3**, all *P <*0.05). Besides, the results of adjusted survival curves also revealed that the cases of parietal pleura invasion with tumor size ≤5cm had close survival outcomes to those with classification pT3 (**Figure 3A**, *P* =0.761). However, they reached much better survival benefits than patients of pT4 classification (adjusted HR =0.743, *P* =0.029). Following adjusting for other confounders, patients for parietal pleura invasion with tumor size >5cm got poorer survival over patients with classification pT3 (adjusted HR =1.532, *P* =0.012), nevertheless, did not have a significant difference with pT4 classification (*P* =0.809).

**Table 3:** Multivariable Cox regression analysis for cancer-specific mortality in patients with different pathological T classifications.

	Multivariable analysis		
Variables	HR	95% CI	P-Value
**Pathological T classification**			
T1	1	reference	
T2	1.593	1.442-1.759	<0.001
T3	2.159	1.876-2.484	<0.001
T4	3.141	2.761-3.573	<0.001
Parietal pleura invasion with tumor size ≤5cm	2.102	1.664-2.656	<0.001
Parietal pleura invasion with tumor size >5cm	2.992	2.269-3.946	<0.001
Rib invasion with any tumor size	3.317	2.718-4.048	<0.001

HR, hazard ratio; CI, confidence interval; NSCLC, non-small cell lung carcinoma.

Cox regression’s method was Enter selection.

Adjust for other confounders, such as sex, age, and the approaches of treatment.

### Prognostic Comparison Between Diseases of Rib Invasion and Pleural Effusion

We analyzed the 96 patients diagnosed with pleura effusion in the SEER database to confirm that the rib invasion was a localized disease as in previous reports. Patients of classification TxN0M1a were included in this study to reduce the effect of lymph node involvement. Only nine patients underwent surgery, and others were diagnosed by other approaches, such as thoracentesis biopsy. The univariable analysis revealed that the patients with pleura effusion had poorer mortality than patients with rib invasion (unadjusted HR =1.983, 95% CI 1.486-2.647, *P* < 0.001, **Figure 3B**).

## Discussion

In the present study, the data of 8681 patients were used to perform principal analysis. The results revealed that parietal pleura invasion and rib invasion were confirmed as independent prognostic risk indicators after adjusting for other confounders. Then, the prognosis of patients with rib invasion had indeed been shown to be worse than that of patients with parietal pleural invasion. Next, a sub-group analysis was conducted in cases with different statuses of chest wall invasion. According to the results of the sub-group analysis, we further re-classify the patients with only parietal pleura invasion into one group for those with tumor size ≤5cm, and another group for those with tumor size over 5cm and put patients with rib invasion in the same group. Based on those classifications, sub-groups of parietal pleura invasion for tumor size ≤5cm and tumor size >5cm got a meaningful stratification effect. The three classifications mentioned above and the classifications pT1, pT2, pT3, and pT4 were conducted to prognostic analysis together. After adjusting for other factors, parietal pleura invasion with tumor size ≤5cm could be considered classification pT3, and parietal pleura invasion with tumor size >5cm might be served as pT4 classification. In addition, rib invasion had the worst survival in that cohort. Parietal pleura invasion plus tumor size ≤5cm is considered pT3; however, patients for parietal pleura invasion plus tumor size >5cm should be treated as pT4. Rib invasion has a poorer prognosis than pT4. Besides, 96 patients with pleural effusion (TxN0M1a classification) were compared with those with rib invasion, which found that the prognosis of pleural effusion was much worse than that of rib invasion. Therefore, we suggested that parietal pleura invasion plus tumor size ≤5cm was treated as pT3; however, parietal pleura invasion plus tumor size with 5.1-7.0cm should ascend to pT4. Those findings may help the treatment plan before surgery. For example, patients diagnosed with NSCLC by biopsy are regarded as early-stage lung cancer only from the tumor size. However, if chest wall invasion is found on imaging, the clinical combined stage may change from early to locally advanced. For patients with locally advanced stages, neoadjuvant therapy may benefit the patients’ complete resection rates (12). Given the prognosis of M1a classification was poorer than rib invasion; hence, rib invasion should continue to be classified as a local disease rather than a metastatic disease. Thus, surgical resection is still an appropriate therapeutic modality for patients with chest wall invasion.

The prognostic difference between the parietal pleura invasion and rib invasion remains unclear. Previous studies majorly paid attention to the role of resection for chest wall on the prognosis of NSCLC patients who had the disease with chest wall invasion (13, 14). In addition, for patients with chest wall invasion who received surgical resection, the extent of nodal involvement (15, 16), the completeness of resection (15, 16), blood transfusion (17), and forced vital capacity (17) were confirmed to be associated with prognosis. Nevertheless, it was controversial whether the depth of invasion affects the prognosis for patients with chest wall invasion. In the studies by Robert J. Downey et al. and Hidehito Matsuoka et al., patients with only parietal pleura invasion did not have a more significant survival improvement, compared with patients with rib invasion (15, 18). The studies from Francesco Facciolo et al. and Alain Chapelier et al. analyzed the data of patients with chest wall invasion, respectively, and got the contrary results that the depth of chest wall involvement impacted the prognosis of those patients (16, 19). The present study results also revealed that rib invasion had a worse survival over the parietal pleura invasion. The survival analyses of studies by Robert J. Downey et al. and Hidehito Matsuoka et al. were not adjusted for other confounders. Therefore, other factors might affect their results, such as age, gender, and differentiation grade. In the clinical practice and TNM staging system, tumor size and differentiation are still essential indicators to confirm the combined stage and to estimate the prognosis (3, 5, 20). Nevertheless, previous studies had neglected to consider the two abovementioned factors when exploring the effect of depth of chest wall invasion on patients’ prognosis (8, 16, 18). In the present study, differentiation grade and tumor size, which were considered factors affecting prognosis, were recognized and adjusted by us. We conclude that after adjusting for these confounding factors, the prognosis of rib invasion is much worse than that of pleural invasion. These findings might provide information for investigating the difference between parietal pleura invasion and rib invasion to The International Association for the Study of Lung Cancer (6).

The role played by parietal pleural invasion and rib invasion in the T classification still needs to be revealed. Although the ^eighth^ edition staging system classified chest wall invasion as descriptor pT3 (4), the effect of depth of chest wall invasion on prognosis had been found (8, 19). As shown in the study by Zhao Mengmeng et al., patients with rib invasion had a worse prognostic trend than other patients, and were finally served as pT4 classification (8). Our study also found that rib invasion might be an independent poor prognostic factor and even had much worse survival than pT4 classification. We also found that the patients with rib invasion prognosis were much better than those with pleural metastasis. Thus, rib invasion could be served as a pT4 descriptor. This also proves that operation is still an important treatment for those patients. In addition, a recent study found that patients with visceral pleura invasion and tumor size of 3.1-4cm should consider re-classification from T2a to T2b (21). The results from the study by Qi M et al. let us note whether patients with parietal pleura invasion will also have a pT-classification ascending performance after combining with the influence of tumor size. Based on this, we performed the sub-group analysis in the cases with parietal pleura invasion. As a result, we revealed that patients with parietal pleura invasion and tumor size with 5.1-7.0cm could be appropriately upstaged from pT3 (stage IIB) to pT4 (modified stage IIIA). Those findings may provide information for accessing prognosis, treatment decisions, and the development of the next new staging system.

This study has several limitations. First, although the data we used were from a large population-based cohort, some important information was not detailed, such as the completeness of resection, the number of resected ribs, and the area where the tumor invaded the chest wall, as we could not obtain it in the SEER database. Second, we did not further categorize the cases of rib invasion because of the small scale of patients. Third, we excluded patients without surgery to ensure that all patients only had localized-invasion disease and did not have pleural metastases. However, this selection criterion caused our study to lose a proportion of patients with chest wall invasion who did not undergo surgery. Of note, it is challenging to identify parietal pleural invasion on imaging. Thus, for patients who do not undergo surgical removal of the tumor, it is not possible to ascertain whether there is pleural involvement or not. Therefore, those shortcomings caused our results to apply only to patients after operation. Finally, given this study belonged to a retrospective study, it was impossible to avoid selection bias. Therefore, more studies are necessary to validate our findings further.

## Conclusions

Rib invasion has a poorer prognosis than pT4. Patients with parietal pleura invasion and tumor size of 5.1-7.0cm could be appropriately up-classified from pT3 to pT4.

## Data Availability Statement

The raw data supporting the conclusions of this article will be made available by the authors, without undue reservation.

## Ethics Statement

The studies involving human participants were reviewed and approved by Ethics committee of Shanghai Pulmonary Hospital and Sun Yat-sen University Cancer Center. The ethics committee waived the requirement of written informed consent for participation.

## Author Contributions

D.X, Z-XL, L-LW, C-WL, and G-WM contributed to the study design, data collection, data analyses, data interpretation, and manuscript drafting. L-LW, L-HQ, S-QX, W-KL, and C-WL contributed to data analyses and manuscript review. W-KL, L-LW, L-HQ, C-WL, G-WM, and DX contributed to data interpretation and manuscript review. All authors contributed to the final approval of the manuscript.

## Funding

This study was supported by Shanghai ShenKang Hospital Development Centre (SHDC22020218), Science and Technology Commission of Shanghai Municipality (21Y11913400), Shanghai Pulmonary Hospital Foundation (fkxr1904), and Outstanding Young Medical Talent of Rising Star in Medical Garden of Shanghai Municipal Health Commission “Dong Xie”. The funding bodies played no role in the design of the study and collection, analysis, and interpretation of data and in writing the manuscript. The funding bodies played a role in the interpretation of data, in writing, and in reviewing the manuscript.

## Conflict of Interest

The authors declare that the research was conducted in the absence of any commercial or financial relationships that could be construed as a potential conflict of interest.

## Publisher’s Note

All claims expressed in this article are solely those of the authors and do not necessarily represent those of their affiliated organizations, or those of the publisher, the editors and the reviewers. Any product that may be evaluated in this article, or claim that may be made by its manufacturer, is not guaranteed or endorsed by the publisher.
